# Evaluation of the advantage of surgeons certified by the endoscopic surgical skill qualification system participating in laparoscopic low anterior rectal resection

**DOI:** 10.1002/ags3.12763

**Published:** 2023-12-14

**Authors:** Naruhiko Sawada, Tomonori Akagi, Manabu Shimomura, Yukitoshi Todate, Kunihiko Nagakari, Hiroaki Takeshita, Satoshi Maruyama, Manabu Takata, Nobuki Ichikawa, Koya Hida, Hiroaki Iijima, Shigeki Yamaguchi, Akinobu Taketomi, Takeshi Naitoh, The EnSSURE Study Group Collaboratives in Japan Society of Laparoscopic Colorectal Surgery., Akinobu Furutani, Akiyoshi Kanazawa, Akiyoshi Noda, Atsushi Ishibe, Chikayoshi Tani, Daisuke Yamamoto, Fumihiko Fujita, Fuminori Teraishi, Fumio Ishida, Fumitaka Asahara, Heita Ozawa, Hideaki Karasawa, Hideki Osawa, Hiroaki Nagano, Hirofumi Ota, Hirokazu Suwa, Hiroki Ochiai, Hiroomi Ogawa, Hiroshi Saeki, Hirotoshi Hasegawa, Hiroyuki Bando, Hisanaga Horie, Hisashi Nagahara, Jun Watanabe, Kaori Hayashibara, Kay Uehara, Kazuhiro Takehara, Ken Kojo, Ken Okamoto, Kenichiro Saito, Koji Ikeda, Koji Munakata, Koki Otsuka, Koki Goto, Mamoru Uemura, Manabu Shiozawa, Manabu Yamamoto, Masaaki Ito, Masafumi Inomata, Masakatsu Numata, Masahiko Watanabe, Masashi Miguchi, Masatsune Shibutani, Mayumi Ozawa, Mitsuhisa Takatsuki, Naoya Aisu, Nobuaki Suzuki, Ryo Ikeshima, Ryo Inada, Ryuichi Oshima, Shigehiro Kojima, Shigenori Homma, Shiki Fujino, Shinichiro Mori, Shinobu Ohnuma, Sho Takeda, Shota Aoyama, Shuji Saito, Shunpei Mukai, Shusaku Takahashi, Takahiro Sasaki, Takahiro Yamanashi, Takeru Matsuda, Takuya Miura, Tatsunari Fukuoka, Tatsunori Ono, Tatsuya Kinjo, Tatsuya Shonaka, Teni Godai, Tohru Funakoshi, Tomohiro Adachi, Tomohiro Yamaguchi, Tomohisa Furuhata, Toshimoto Kimura, Toshisada Aiba, Toshiya Nagasaki, Toshiyoshi Fujiwara, Tsukasa Shimamura, Tsunekazu Mizushima, Yasuhito Iseki, Yasuo Sumi, Yasushi Rino, Yasuyuki Kamada, Yohei Kurose, Yoshiaki Kita, Yoshihiro Kakeji, Yoshihiro Takashima, Yoshihito Ide, Yoshiharu Sakai, Yoshinori Munemoto, Yoshito Akagi, Yoshiyuki Ishii, Yuji Inoue, Yuki Kiyozumi, Yukihito Kokuba, Yusuke Suwa, Yusuke Sakimura, Yusuke Shimodaira

**Affiliations:** ^1^ Digestive Disease Center Showa University Northern Yokohama Hospital Yokohama Japan; ^2^ Department of Gastroenterological and Pediatric Surgery Oita University Oita Japan; ^3^ Hiroshima City North Medical Center Asa Citizens Hospital Hiroshima Japan; ^4^ Department of Surgery Southern Tohoku General Hospital Koriyama Japan; ^5^ Department of Digestive and General Surgery Juntendo University Urayasu Hospital Urayasu Japan; ^6^ Department of Surgery National Hospital Organization Nagasaki Medical Center Nagasaki Japan; ^7^ Department of Gastroenterological Surgery Niigata Cancer Center Hospital Niigata Japan; ^8^ Department of Surgery Nagano Municipal Hospital Nagano Japan; ^9^ Department of Gastroenterological Surgery I, Graduate School of Medicine Hokkaido University Sapporo Japan; ^10^ Department of Surgery Kyoto University Hospital Kyoto Japan; ^11^ Division of Colorectal Surgery, Department of Surgery Tokyo Women's Medical University Tokyo Japan; ^12^ Department of Lower Gastrointestinal Surgery Kitasato University School of Medicine Sagamihara Japan

**Keywords:** endoscopic surgical skill qualification system, laparoscopic low anterior resection, rectal cancer

## Abstract

**Background:**

A technical qualification system was developed in 2004 by the Japan Society for Endoscopic Surgery. An analysis of the EnSSURE study on 3188 stage II–III rectal cancer patients, which was performed by including the participation of qualified surgeons as assistants and advisers without restricting their participation as operators, revealed that the participation of technically qualified surgeons in surgery improved the technical and oncological safety of laparoscopic rectal resection.

**Aim:**

This secondary retrospective analysis of the EnSSURE study examined the advantage of qualified surgeons participating in laparoscopic low anterior resection (LAR).

**Methods:**

The outcomes of low anterior resection were compared between groups with and without the participation of surgeons qualified by the Endoscopic Surgical Skill Qualification System (*Q* and non‐*Q* groups, respectively). We used propensity score matching to generate paired cohorts at a one‐to‐one ratio. The postoperative complication rate, short‐term results (hemorrhage volume, operative time, number of dissected lymph nodes, open conversion rate, intraoperative complication rate, and R0 resection rate), and long‐term results (disease‐free survival rate, local recurrence rate, and overall survival rate) were evaluated.

**Results:**

The frequencies of postoperative complications, anastomotic bleeding, and intraperitoneal abscess were significantly lower, the operative time was significantly shorter, the postoperative hospital stay was significantly shorter, and the number of dissected lymph nodes was higher in the *Q* group. No significant differences were observed in disease‐free survival, local recurrence, or overall survival rate rates between the groups.

**Conclusion:**

The participation of qualified surgeons in LAR is technically advantageous.

## Graphical abstract

A technical qualification system was developed in 2004 by the Japan Society for Endoscopic Surgery, and an analysis of the EnSSURE study revealed that the participation of technically qualified surgeons in surgery improved the technical and oncological safety of laparoscopic rectal resection. Our secondary retrospective analysis of the EnSSURE study examined the advantages of qualified surgeons participating in laparoscopic low anterior resection. The participation of qualified surgeons in LAR was shown to be technically advantageous.

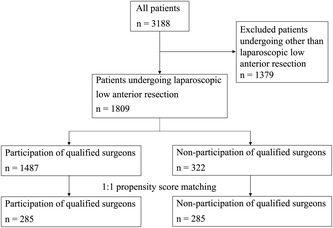

## 1 INTRODUCTION

Colorectal cancer affects 1 million individuals worldwide each year, and its prevalence is the highest among all cancers in Japan.^1^ Laparoscopic colon resection was initially performed by Watanabe et al.^2^ in Japan in 1992. The Japan Society for Endoscopic Surgery established a technical qualification system in 2004 that certifies surgeons qualified to have surgical skill and teach and train young surgeons. Surgeons who meet standards for experience and performance submit unedited videos of surgery, and their technical proficiency is evaluated and rated by two or more judges. The technical qualification system is considered to have contributed to the handing down of skills and securing the quality of laparoscopic colon resection. Although the beneficial effects of the technical qualification system have been reported,^3^, ^4^ it has not yet been examined in a multicentric large‐scale study, and, thus, the bibliographic basis is considered to be deficient. In an analysis of 1428 cases of surgery performed at 11 institutions between 2010 and 2013 by Ichikawa et al.,^5^ long‐term results indicated that the qualification system contributed to a reduction in the local recurrence rate of stage II colorectal cancer. Based on these findings, the participation of qualified surgeons in surgery, particularly that for rectal cancer, is considered to improve the short‐term results achieved by surgeons in the training stage in average hospitals. Akagi et al.^6^ suggested that surgery performed by qualified surgeons reduces the risk of anastomotic leakage in laparoscopic low anterior resection (LAR). Furthermore, the direct handling of surgery by a qualified surgeon appeared to effectively shorten the operative time for laparoscopic LAR; however, it did not affect the rate of complications.^3^, ^4^, ^7^

Ichikawa et al.^8^ conducted an analysis of a large‐scale study (EnSSURE study) on 3188 stage II–III rectal cancer patients who underwent surgery between 2014 and 2016 at 56 institutions participating in the Japan Society of Laparoscopic Colorectal Surgery. The findings obtained showed that the participation of qualified surgeons (one or more qualified surgeons participated as the operator or supervisor), compared with resections without their participation, improved the technical and oncological safety of laparoscopic rectal resection in Japan. These findings demonstrated the usefulness of this certification system because one of its purposes is to qualify the competency of the candidate as a supervisor. However, this study did not focus on differences among the different types of procedures (such as high anterior resection, LAR, or Mile's procedure) and did not evaluate the impact of the attendance of a qualified surgeon on each procedure.

The present study is a secondary retrospective analysis of the EnSSURE study. We investigated whether the participation of qualified surgeons in surgery improved technical and oncological safety by restricting the surgical procedure to laparoscopic LAR, which was the most frequently performed procedure in the EnSSURE study.

## 2 MATERIALS AND METHODS

Patients who underwent laparoscopy‐assisted LAR for rectal cancer between January 2014 and December 2016 at 56 institutions were retrospectively analyzed. Subjects were patients histologically diagnosed with adenocarcinoma primarily affecting RS to Rb, those who underwent LAR, those with stage II or III disease, those who underwent elective surgery, and those who agreed to participate in this study. Exclusion criteria were as follows: (1) patients with multiple/double cancer (those with a disease‐free period ≤5 years were included in double cancers; those with cancers in situ were not excluded), (2) those who simultaneously underwent other surgeries, (3) those who underwent robot‐assisted surgery, (4) those with ulcerative colitis (colitic cancer), and (5) those judged by an investigator to be inappropriate as subjects.

The internal review committees of Hokkaido University Hospital (No. 019‐0328) and all participating hospitals approved this study as exempt human subject research, and informed consent was obtained by the opt‐out method in accordance with the guidelines of the Japanese Ministry of Health, Labour, and Welfare. This study was registered with the UMIN Clinical Trials Registry System on June 3, 2020 (UMIN 000040645).

**FIGURE 1:**
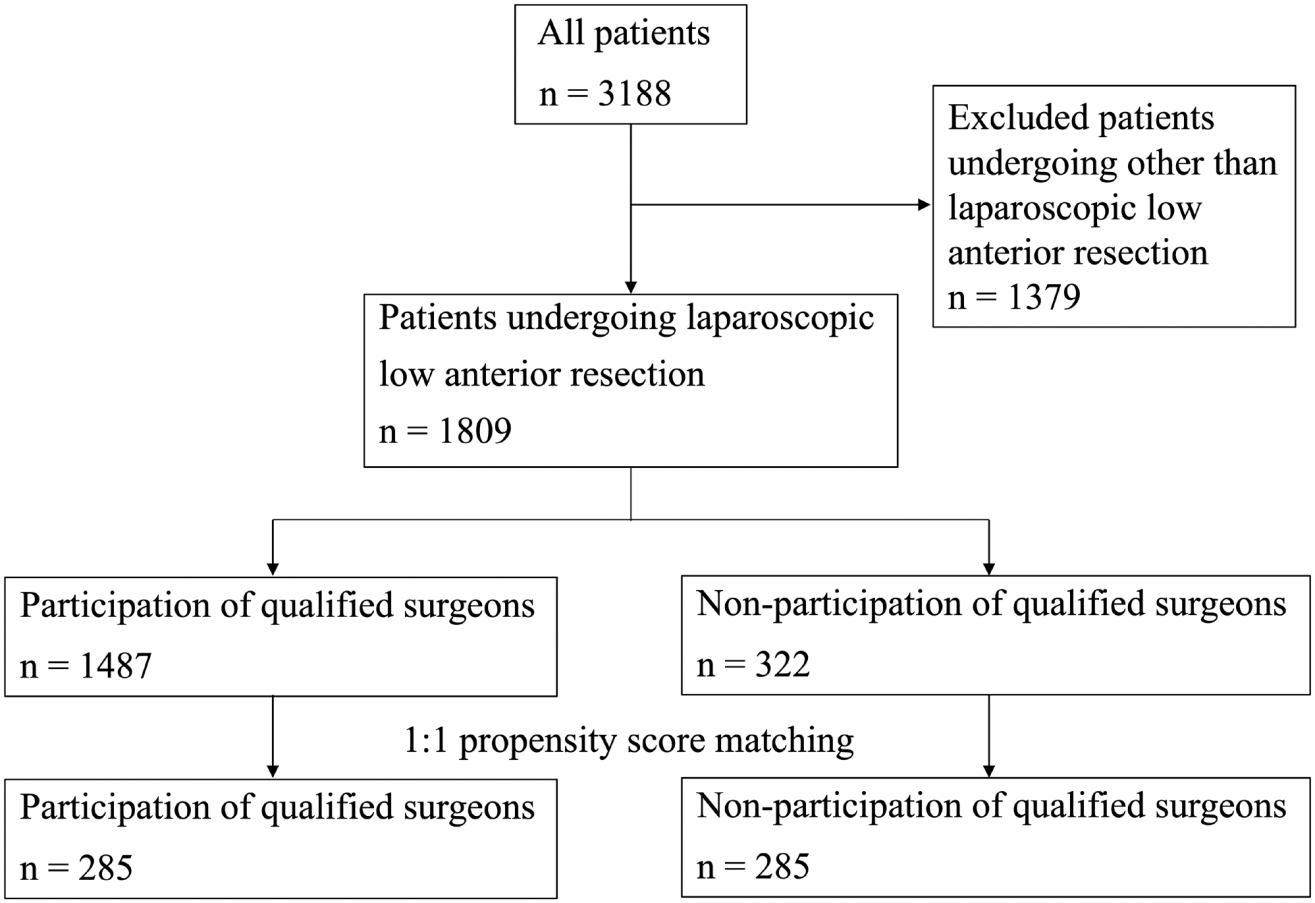
Patient flow chart. Parameters used in propensity score matching are: age, sex, body mass index (BMI), the American Society of Anesthesiologists (ASA) class, obstruction, the clinical T stage (cT), clinical N stage (cN), and tumor location, preoperative therapy, procedures, combined resection of the surrounding organs, lymph node dissection (D1, D2, or D3), lateral pelvic node dissection, high ligation of the inferior mesenteric artery (IMA), mobilization of the splenic flexure, a diverting stoma, and the type and volume of the institution.

The outcomes of LAR were compared between the groups in which surgeons qualified by the Endoscopic Surgical Skill Qualification System (ESSQS) participated or not in surgery (the *Q* and non‐*Q* groups), respectively. The backgrounds of the two groups were equalized by propensity score matching (PSM). The details of parameters are provided in the statistical analysis section. A patient flow diagram is shown in Figure 1.

### 2.1 Endpoints

As the primary endpoint, the rate of postoperative complications (≥Grade III by the Clavien–Dindo classification) was evaluated. As the secondary endpoints, (1) short‐term results (hemorrhage volume, operative time, postoperative hospital stay, the rate of anastomotic bleeding, intraperitoneal bleeding, intraperitoneal abscess, wound infection, obstruction, anastomotic leakage and reoperation, the number of dissected lymph nodes, open conversion rate, intraoperative complication rate, and R0 resection rate) and (2) long‐term results (recurrence‐free survival, local recurrence rate (LRR), and overall survival (OS) rates) were assessed.

### 2.2 Statistical analysis

Continuous data were reported as the mean ± standard deviation. Categorical data were statistically tested by the chi‐square test and continuous data by the Student's *t*‐test. Clinical and surgical features, including age, sex, body mass index (BMI), the American Society of Anesthesiologists (ASA) class, obstruction, the clinical T stage (cT), clinical N stage (cN), tumor location, preoperative therapy, procedures, combined resection of the surrounding organs, lymph node dissection (D1, D2, or D3), lateral pelvic node dissection, high ligation of the inferior mesenteric artery (IMA), mobilization of the splenic flexure, a diverting stoma, and the type and volume of the institution were selected to generate propensity scores. These factors were selected because of their potential impact on surgical outcomes and the decision regarding whether the qualified surgeon participated in the procedures. Propensity scores were generated using logistic regression by setting the outcome as the participation of qualified surgeons. PSM with a 1:1 ratio of the nearest available match was performed using a caliper of 0.1.

Disease‐free survival (DFS) was defined as the time from surgery to the first recurrence (local or distant) of the tumor in months, LRR as the time from surgery to the first local recurrence in months, and OS as the time from surgery to death due to any cause in months. DFS, LRR, and OS were assessed using the Kaplan–Meier survival analysis and compared by the log‐rank test.

A *p* value of <0.05 was considered to be significant. All statistical analyses were performed using JMP® Pro (version 16.0.0; SAS Institute Inc., Cary, NC; 2021).

## 3 RESULTS

Among 3188 cases 1809 underwent LAR. Patient backgrounds are shown in Table 1. Patient backgrounds were equalized by 1:1 PSM, after which no significant differences were observed in pairwise comparisons of covariables (Table 1).

**TABLE 1:** Patient backgrounds before and after matching.

	Patient backgrounds before matching			Patient backgrounds after matching		
	*Q* (*n* = 1487)	Non‐*Q* (*n* = 322)	*p*‐value	*Q* (*n* = 285)	Non‐*Q* (*n* = 285)	*p*‐value
Age (years)*	63.5 (11.5)	64.1 (11.7)	0.37	64.0 (11.4)	63.8 (11.7)	0.79
Sex ratio (M:F)	953:534	204:118	0.80	184:101	183:102	0.93
BMI (kg/m^2^)*	22.6 (3.4)	22.7 (3.8)	0.75	22.8 (3.7)	22.7 (3.7)	0.80
ASA			0.003			0.83
1	474 (31.9)	99 (30.8)		71 (24.9)	68 (23.9)	
2	888 (59.7)	194 (60.3)		190 (66.7)	189 (66.3)	
3	92 (6.2)	29 (9.0)		24 (8.4%)	28 (9.8)	
4	1 (0.1)	0 (0.0)		–	–	
Unknown	32 (2.1)	0 (0.0)		–	–	
Obstruction	72 (4.8%)	16 (5.0%)	0.92	5 (1.8)	11 (3.9%)	0.12
Neoadjuvant therapy			<0.001			0.88
CT	152 (10.2)	11 (3.4)		13 (4.7)	11 (3.9)	
CT + CRT	50 (3.4)	6 (1.9)		5 (1.8)	6 (2.1)	
CRT	126 (8.5)	23 (7.1)		24 (8.4)	20 (7.0)	
No	1159 (77.9)	282 (87.6)		243 (85.3)	248 (87.0)	
Location			0.01			0.90
RS	313 (21.1)	83 (25.8)		67 (23.5)	71 (24.9)	
Ra	754 (50.7)	174 (54.0)		155 (54.4)	150 (52.6)	
Rb	417 (28.0)	65 (20.2)		63 (22.1)	64 (22.5)	
Unknown	3 (0.2)	0 (0.0)		–	–	
Stage			0.67			0.78
II	640 (43.0)	146 45.3)		136 (47.7)	134 (47.0)	
IIIa	603 (40.6)	122 (37.9)		109 (38.3)	105 (36.8)	
IIIb	244 (16.4)	54 (16.8)		40 (14.0)	46 (16.1)	
LND			0.31			0.44
D0	3 (0.2)	0		0	0	
D1	1 (0.1)	1 (0.1)		0	1 (0.4)	
D2	115 (7.7)	19 (5.9)		21 (7.4)	18 (6.3)	
D3	1368 (92.0)	302 (93.8)		264(92.6)	266 (93.3)	
LLND			<0.001			0.95
None	1236 (83.1)	301 (93.5)		263 (92.3)	264 (92.6)	
Unilateral	63 (4.2)	5 (1.6)		6 (2.1)	5 (1.8)	
Bilateral	188 (12.6)	16 (5.0)		16 (5.6)	16 (5.6)	
Diverting stoma	648 (43.6)	116 (36.0)	0.01	114 (40.0)	113 (39.7)	0.93

*Note*: Values in parentheses are percentages unless indicated otherwise; values are *mean (SD).

### 3.1 Postoperative complications

The frequencies of postoperative complications (10.5 vs. 17.9%, *p* = 0.01), anastomotic bleeding (0.00 vs. 1.8%, *p* = 0.008), and intraperitoneal abscess (0.4 vs. 2.5%, *p* = 0.02) were significantly lower in the *Q* group. No significant differences were observed in the frequencies of intraperitoneal bleeding (0.00 vs. 0.70%, *p* = 0.01), wound infection (0.70 vs. 0.70%, *p* = 1.00), obstruction (2.5 vs. 3.2%, *p* = 0.61), or reoperation (4.6 vs. 6.7%, *p* = 0.27) between the two groups (Table 2). The frequency of anastomotic leakage was 9.5% in the non‐*Q* group but was slightly lower at 6.0% in the *Q* group (*p* = 0.12).

**TABLE 2:** Complications.

	*Q* (*n* = 285)	Non‐*Q* (*n* = 285)	*p*‐value
Postoperative complications	30 (10.5)	51 (17.9)	0.01
Anastomotic bleeding	0 (0.0)	5 (1.8)	0.008
Intraperitoneal bleeding	0 (0.0)	2 (0.7)	0.10
Intraperitoneal abscess	1 (0.4)	7 (2.5)	0.02
Wound infection	2 (0.7)	2 (0.7)	1.00
Obstruction	7 (2.5)	9 (3.2)	0.61
Anastomotic leakage	17 (6.0)	27 (9.5)	0.12
Reoperation	13 (4.6)	19 (6.7)	0.27

*Note*: Values in parentheses are percentages.

### 3.2 Operation

The operative time was significantly shorter (277.9 vs. 313.2 min, *p* < 0.001) and the number of dissected lymph nodes was higher (20.6 vs. 18.2, *p* = 0.01) in the *Q* group. The postoperative hospital stay was significantly shorter in the *Q* group than in the non‐*Q* group (16.1 vs. 18.8 days, *p* = 0.03). No significant differences were observed in blood loss (53.8 vs. 70.6 mL, *p* = 0.23), conversion (1.8 vs. 2.8%, *p* = 0.40), intraoperative complications (1.40 vs. 1.8%, *p* = 0.74), or R0 resection (98.6 vs. 97.5%, *p* = 0.28) between the two groups (Table 3).

**TABLE 3:** Operation.

	*Q* (*n* = 285)	Non‐*Q* (*n* = 285)	*p*‐value
Blood loss (mL)*	53.8 (170.0)	70.6 (164.9)	0.23
Operative time (min)*	277.9 (90.2)	313.2 (120.4)	<0.001
Number of dissected lymph nodes*	20.6 (11.0)	18.2 (10.7)	0.01
Conversion	5 (1.8)	8 (2.8)	0.40
Intraoperative complications	4 (1.4)	5 (1.8)	0.74
R0 resection	281 (98.6)	278 (97.5)	0.28
Postoperative hospital stay (days)*	16.1 (11.5)	18.8 (18.0)	0.03

*Note*: Values in parentheses are percentages unless indicated otherwise; values are *mean (s.d.).

### 3.3 Long‐term outcomes

**FIGURE 2:**
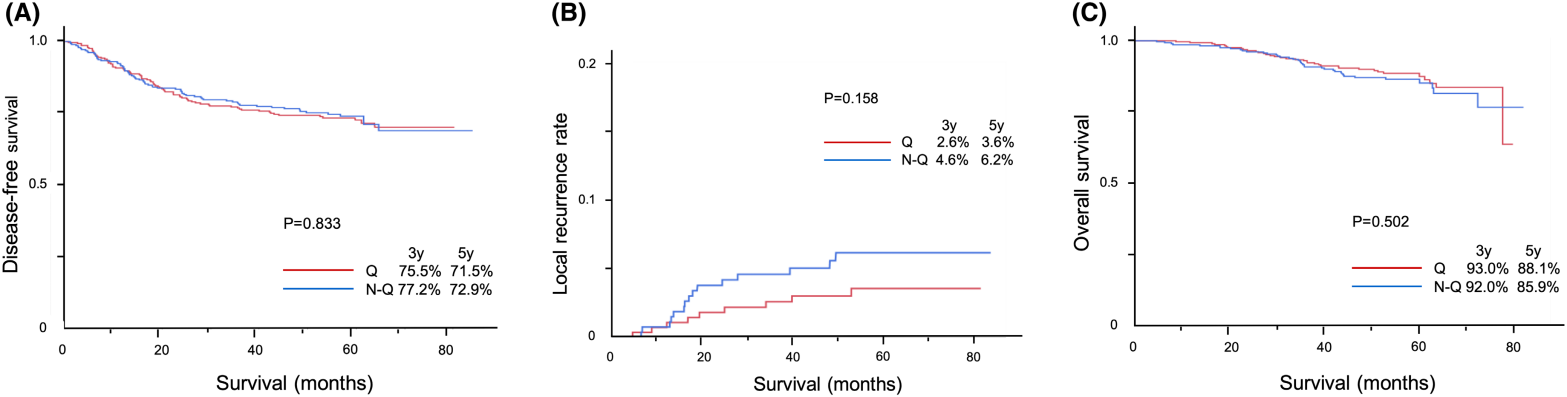
Survival after LAR. (A) Disease‐free survival after LAR. (B) Local recurrence rate after LAR. (C) Overall survival after LAR.

Five‐year DFS rates were 71.5 vs. 72.9%, 5‐year LR rates were 3.6 vs. 6.2%, and 5‐year OS rates were 88.1 vs. 85.9% in the *Q* and non‐*Q* groups, respectively. No significant differences were observed in DFS (*p* = 0.833), LRR (*p* = 0.158), or OS (*p* = 0.502) rates between the two groups (Figure 2).

## 4 DISCUSSION AND CONCLUSION

We investigated whether the participation of certificated surgeons in surgery improved technical and oncological safety by restricting the surgical procedure to laparoscopic LAR, and demonstrated that the frequencies of severe postoperative complications, anastomotic bleeding, and intraperitoneal abscess were significantly lower, the operative time and postoperative hospital stay were significantly shorter, and the number of dissected lymph nodes was higher in surgery with a certificated surgeon. The present results indicate the technical advantage of the participation of ESSQS‐qualified surgeons in laparoscopic LAR for rectal cancer.

Laparoscopic surgery performed by inexperienced but well‐trained surgeons may achieve similar surgical results to that by experienced surgeons.^9^, ^10^, ^11^, ^12^ Outside Japan, there are training centers for laparoscopy, such as the IRCAD Laparoscopic Training Center (Strasbourg, France),^8^ World Laparoscopy Training Institute (Florida, USA), and World Laparoscopy Hospital (Delhi, India), which collect students from all over the world. In addition, in the United States, surgeons who fail to meet the standards required by the Fundamentals of Laparoscopic Surgery are not permitted to perform endoscopic surgery.^13^ The UK also has a training program for laparoscopic surgery, which contributes to improvements in the skill of laparoscopy.^14^, ^15^ However, Japan has no standard program for training or a qualification system for laparoscopic surgery.^16^ The environment for the training of learners of laparoscopy that offers classroom sessions and practice using animals and dry boxes is considered to be more advanced than in Japan. However, a qualification system based on the evaluation of operation videos on actual patients as the result of such training has not yet been introduced. The establishment of a skill qualification system using actual operation videos by the Japan Society for Endoscopic Surgery was epoch‐making. Although there is a training program for laparoscopic surgery in Thailand,^17^ efforts to introduce a technical qualification system for laparoscopic surgery similar to that of Japan have been initiated.

Eighteen years have passed since the establishment of ESSQS. This system aims to promote the sound dissemination and development of endoscopic surgery in Japan and also to contribute to the well‐being of Japanese individuals. However, there are a number of limitations, such as the time and effort needed to acquire certification as a qualified surgeon and the assignment of cases to particular surgeons for them to acquire the certification with resultant decreases in opportunities for other surgeons to perform surgery. Furthermore, surgeons must pass a difficult technical examination, in which only 20–30% of candidates are successful. Studies are needed to confirm whether certification by this system is worth the time and effort, and a multicenter large‐scale study was not performed until that by Ichikawa et al. According to the analysis by Ichikawa et al.,^8^ short‐term results, such as the complication rate, were confirmed to be improved by the participation of a qualified surgeon in all cohorts, indicating the usefulness of the qualification system. Qualified surgeons are evaluated based on the results of laparoscopy‐assisted high anterior resection; however, in the present study, we evaluated the short‐ and long‐term results of LAR with an emphasis on long‐term results because we considered it necessary to evaluate the advantages of the qualification system for other procedures.

In LAR, the qualification system was shown to be useful for reducing complications and shortening the operative time, which was consistent with the results of the analysis of all cohorts, and the difference between qualified and unqualified surgeons became evident. Difficult surgical procedures are, in reality, a series of basic techniques, and surgeons are required to accomplish surgery through their effective combination. The qualification system is considered to properly assess the basic techniques needed to safely perform surgery. Therefore, the qualification system of surgeons is considered satisfactory in that it appropriately assesses skill levels based on minute differences. In the present study, a difference was observed in the postoperative hospital stay, which may have been due to postoperative complications. The participation of qualified surgeons is also important for reducing medical costs.

As for anastomotic bleeding, we think that possible reasons for anastomotic hemorrhage in non‐certified physicians may be that mesorectum may not be sufficiently dissected during skeletonization of the rectum or that the mesorectum may have been pulled during anastomosis. As for intraperitoneal abscess, this can be caused by minor leaks. Leaks may have resulted from anastomotic tension due to insufficient passivity, or inadequate blood flow due to failure of mesenteric ligation in the non‐certified surgeons.

Although a previous study showed that the presence or absence of ESSQS‐qualified surgeons was independently related to local recurrence in patients with stage II disease,^5^ another study reported no difference in the 3‐year recurrence‐free survival rate.^3^ In the present study, no significant differences were noted in DFS, LRR, or OS. These results suggest that the participation of a qualified surgeon in surgery is preferable; however, if this is impossible, laparoscopic surgery is permissible in an environment that ensures the safe implementation of surgery that may include the participation of a qualified surgeon as an advisor or instructor because we consider that the fundamental quality of laparoscopic rectal surgeries has progressed to adequate levels nationwide.

Robot‐assisted surgery for rectal cancer has recently been increasing.^18^, ^19^ Since it was excluded from the present study, further studies are needed to evaluate the role of qualified surgeons in robot‐assisted surgery. In conclusion, the technical advantage of the participation of ESSQS‐qualified surgeons in LAR was confirmed.

## 5 COLLABORATERS

EnSSURE study group: Akinobu Furutani, Akiyoshi Kanazawa, Akiyoshi Noda, Atsushi Ishibe, Chikayoshi Tani, Daisuke Yamamoto, Fumihiko Fujita, Fuminori Teraishi, Fumio Ishida, Fumitaka Asahara, Heita Ozawa, Hideaki Karasawa, Hideki Osawa, Hiroaki Nagano, Hirofumi Ota, Hirokazu Suwa, Hiroki Ochiai, Hiroomi Ogawa, Hiroshi Saeki, Hirotoshi Hasegawa, Hiroyuki Bando, Hisanaga Horie, Hisashi Nagahara, Jun Watanabe, Kaori Hayashibara, Kay Uehara, Kazuhiro Takehara, Ken Kojo, Ken Okamoto, Kenichiro Saito, Koji Ikeda, Koji Munakata, Koki Otsuka, Koki Goto, Mamoru Uemura, Manabu Shiozawa, Manabu Yamamoto, Masaaki Ito, Masafumi Inomata, Masakatsu Numata, Masahiko Watanabe, Masashi Miguchi, Masatsune Shibutani, Mayumi Ozawa, Mitsuhisa Takatsuki, Naoya Aisu, Nobuaki Suzuki, Ryo Ikeshima, Ryo Inada, Ryuichi Oshima, Shigehiro Kojima, Shigenori Homma, Shiki Fujino, Shinichiro Mori, Shinobu Ohnuma, Sho Takeda, Shota Aoyama, Shuji Saito, Shunpei Mukai, Shusaku Takahashi, Takahiro Sasaki, Takahiro Yamanashi, Takeru Matsuda, Takuya Miura, Tatsunari Fukuoka, Tatsunori Ono, Tatsuya Kinjo, Tatsuya Shonaka, Teni Godai, Tohru Funakoshi, Tomohiro Adachi, Tomohiro Yamaguchi, Tomohisa Furuhata, Toshimoto Kimura, Toshisada Aiba, Toshiya Nagasaki, Toshiyoshi Fujiwara, Tsukasa Shimamura, Tsunekazu Mizushima, Yasuhito Iseki, Yasuo Sumi, Yasushi Rino, Yasuyuki Kamada, Yohei Kurose, Yoshiaki Kita, Yoshihiro Kakeji, Yoshihiro Takashima, Yoshihito Ide, Yoshiharu Sakai, Yoshinori Munemoto, Yoshito Akagi, Yoshiyuki Ishii, Yuji Inoue, Yuki Kiyozumi, Yukihito Kokuba, Yusuke Suwa, Yusuke Sakimura, Yusuke Shimodaira.

## AUTHOR CONTRIBUTIONS

Conceptualization: NS, TA, NI, KH, HI, SY, AT, TN. Data curation: MS, YT, KN, HT, SM, MT, NI, HI. Formal analysis: NS, NI, HI. Investigation: NS, TA, MS, YT, KN, HT, SM, MT, NI, KH. Project administration: TA, NI, AT, AT, TN. Supervision: TA, NI, KH, HI, SY, AT, TN. Writing – original draft: NS. Writing ‐ review & editing: TA, MS, YT, KN, HT, SM, MT, NI, KH, HI, SY, AT, TN.

## FUNDING INFORMATION

The authors have no funding to declare.

## CONFLICT OF INTEREST STATEMENT

The authors declare no conflict of interest.

## ETHICS STATEMENT

Informed Consent: N/A.

Registry and the Registration No. of the study/trial: N/A.

Animal Studies: This study was conducted according to the principles of the Declaration of Helsinki. The internal review committee of the Hokkaido University Hospital (No. 019‐0328) and all participating hospitals approved this study as exempt human subject research, and informed consent was obtained by the opt‐out method in accordance with the guidelines of the Japanese Ministry of Health, Labor, and Welfare.

## Data Availability

The datasets generated during and/or analyzed during the current study are available from the corresponding author on reasonable request.
